# Publishing confirming and non-confirming data

**DOI:** 10.12688/f1000research.7847.1

**Published:** 2016-02-04

**Authors:** Bruce Alberts, Alexander Kamb

**Affiliations:** 1 Department of Biophysics and Biochemistry, University of California, San Francisco, CA, USA; 2 Discovery Research, Amgen Inc., Thousand Oaks, CA, USA

**Keywords:** Reproducibility, Robustness, Amgen

## Abstract

This editorial introduces the Preclinical Reproducibility and Robustness channel on *F1000Research*, which has been created to encourage and facilitate open and transparent publication and discussion of confirmatory and non-confirmatory studies in biomedical research.

## Editorial

In 2012 Begley and Ellis shocked the academic community by reporting that scientists at Amgen, a major biotech company, could not replicate the findings of nearly 90% of 53 high-profile oncology publications ^1^. This study followed other, less publicized disclosures questioning the reliability of conclusions in the biomedical literature ^2– 5^. Today, scientific leaders, patient advocates—even economists—are coming forward in greater numbers to challenge the quality and efficiency of medical research. New efforts have begun to explicitly repeat a sample of the research reported in high-profile publications. For the psychology field, a recent paper in *Science* magazine reports that "a large portion of replications produced weaker evidence for the original findings" ^6^, and a contract research organization has been funded to begin a systematic effort to reproduce experiments from 50 high-impact oncology publications ^7^. We write to promote an additional effort to improve scientific standards--one aimed at strengthening the self-correcting nature of science through the widespread, rapid publication of the failures (as well as the successes) of attempts to reproduce published scientific findings.

Scientific publication has long been the critical mechanism for conveying scientific data to the world, allowing the conclusions of one team of scientists to be tested by others and, if confirmed, to be extended into the unknown. The model of nature that science has erected is thereby built up detail by detail, layer upon layer, on a foundation of self-consistent logic and experiment. Because science depends on observations that are verifiable, science is at its core self-correcting. But the process of self-correction in science must be improved. Today, many intriguing, but non-robust conclusions that remain unchallenged in the biomedical literature create opportunity costs for drug development, forcing both the biopharmaceutical industry and academic scientists to devote major resources to validating, rather than extending, results.

The vast majority of scientists are well-intentioned, and they want to achieve, discover and invent. They strive to uncover the truth about nature and to get the credit for doing so. But science is a human endeavor. Although intellect, diligence, aspiration and passion drive inexorable progress, ego and unconscious bias are also involved. All scientists have encountered non-robust findings in the literature. We know that deliberate fraud is rare, but wishful thinking is common, and potentially dangerous.

Writers, readers, reviewers, granters, and editors all prefer a good story. This automatically introduces a bias into the publication process favoring positive results. Non-confirming data are communicated much less frequently and effectively than are provisional, positive results. For this reason, energetic steps must be taken to make the publication of scientific results more balanced. Scientists must create new paths and rewards that lead to much more efficient, rapid publication of failures to reproduce published results. The scientific community urgently requires unobstructed visibility of non-confirming datasets, with clear expositions of the materials used and experiments conducted. Scientists can then compare the datasets to form their own conclusions. We cannot require perfection prior to publication, which would be at best impractical, and at worst disastrous. But we can make the inevitable imperfections easier to identify.

Industry can enhance the self-correcting nature of science by organizing a robust effort to publish both its non-confirming and its confirming results, while simultaneously encouraging publication of such validation experiments by academic scientists. This editorial announces the creation of a special online channel on the open science platform *F1000Research* that will be dedicated to publications of non-confirming and confirming results. The Preclinical Reproducibility and Robustness channel will focus on thorough reporting of the methods used, and ensure access to the source data underlying the findings. Amgen scientists have just published three such research articles on this channel as an initial effort to stimulate this process of science self-evaluation.

Each submission to this channel will undergo a fully transparent post-publication peer review following the *F1000Research* publication model. Referee reports from invited named experts will be posted alongside the article, allowing readers to get a full picture of the soundness of the validation experiments. The original authors can provide signed comments on the article, or publish their own full Correspondence article (for peer review) in the channel if they have further (published or new evidence) that adds to the discussion. It is our hope that, both through this format and others, a vigorous new publishing culture can be established to enhance the crucial self-correcting feature of science.
